# Liposome-Based Flow Injection Immunoassay System

**DOI:** 10.6028/jres.093.160

**Published:** 1988-12-01

**Authors:** Laurie Locascio-Brown, Anne L. Plant, Richard A. Durst

**Affiliations:** Center for Analytical Chemistry, National Institute of Standards and Technology, Gaithersburg, MD 20899

The development of many biochemical assays is dependent upon the specific interaction of an antigen with its antibody. This interaction is usually monitored using secondary labels with the most popular immunoassays employing fluorescent or radioactive tracers for detection of a single binding event. We are developing a novel flow injection analysis (FIA) system which contains an immunospecific reactor column and utilizes liposomes for detection. The fluorophore-loaded liposomes used in this assay can be made to provide signal enhancement in the range of one thousand to one million times per binding event making fluorescent assays competitive in sensitivity with radioimmunoassays.

Liposomes are spherical structures composed of phospholipid bilayers that form spontaneously when phospholipid molecules are dispersed in water. The interior and exterior environments of liposomes are aqueous and, therefore, liposomes can be prepared with large numbers of water-soluble marker molecules trapped in their internal aqueous space. Liposomes are prepared by the injection method [1] from a mixture of dimyristoylphos-phatidylcholine: cholesterol: dicetyl phosphate with a molar ratio of 5:4:1. Approximately 1 × 10^3^ carboxyfluorescein molecules are encapsulated inside each liposome when the liposomes are formed in 3 mmol/L carboxyfluorescein solution. The liposomes may be “sensitized” to a particular antigen through covalent binding of that antigen to the polar head group of a phospholipid molecule which is incorporated into the lipid mixture at about 1 mol % prior to liposome formation. When the liposome is formed in water, approximately half of the antigen will be exposed to the external solution, and, therefore, will be available to interact with antibody binding sites. The combining of antigen on these “sensitized’ liposomes with antibody can then be monitored through the fluorescence of the encapsulated marker molecule. In this assay, each binding event is amplified approximately 1000 times. Liposomes containing 3 mmol/L carboxyfluorescein are about 0.1 *μ*m in diameter, and have a polydispersity of 0.1 which corresponds to a size distribution of 0.5% as determined by photon correlation spectroscopy with data analysis using the cumulants method [2]. The diffusion coefficient of these liposomes in water was determined to be 5 × 10^−8^ cm^2^/s, and the liposomes contained 1–5 lamellae per liposome. These liposomes have been determined to be stable for greater than 3 months in TRIS buffer at room temperature.

The flow injection system contains a glass reaction column (2.0 mm i. d. × 99.5 mm; total volume of 155 *μ*L) packed with nonporous glass beads 250±40 *μ*m in diameter (60–80 mesh) which provide the solid phase for the immuno-specific reaction. The antibody is covalently bound to the solid support in a manner which ensures the proper orientation of the antibody binding site such that there is very little loss in activity. The antibody is prepared for binding by initially cleaving the Fc portion of the antibody with pepsin leaving an F(ab′)_2_ fragment attached in the hinge region through a disulfide bond. Reducing this disulfide bond yields two Fab′ fragments with equal affinity for antigen binding and with a sulfhydryl group at the base of the fragment available for attachment. Covalent immobilization of this Fab′ fragment is achieved through silanization of the beads with an aminosilane reagent. An amide bond is formed between the aminosilane and a difunctional succinimidyl-maleimido linking agent. The Fab′ fragment is then easily attached to the maleimido group through the sulfhydryl group at the base of the antibody fragment (fig. 1). Several preparations of nonporous soda lime glass beads derivatized with a monofunctional silane have proven to be stable in a packed column for 7 d when subjected to a flow rate of 1 mL/min in slightly basic TRIS buffer. Quantitation of silanized sites is accomplished through acetylation of the amino group using ^14^C-labeled acetic acid. Detection of the interaction between sample antigen (analyte) molecules, and the covalently immobilized antibodies is achieved by competition with liposomes that are “sensitized” with antigen.

The flow properties of liposomes in flow injection systems were investigated to determine optimal conditions for competitive immunochemical binding events in a heterogeneous sample. It was found that due to the large differences in diffusion coefficients of liposomes and small solution molecules, some separation of the sample and liposome solution boluses occurred prior to the introduction of the mixture onto the column. This precolumn separation reduces the sensitivity of the assay since the arrival of the liposome bolus precedes the sample bolus, and many sites become saturated before any competition can take place. Precolumn mixing of the liposome suspension reduces the separation of the boluses, and a simple solution to the problem was found in simultaneous separate injection of the liposome-antigens and free antigens. The liposome-antigens were injected using a knitted sample loop while sample solutions were injected through a straight sample loop [3] as shown in figure 2. This manipulation was sufficient to cause significant precolumn bolus overlap, and allowed the full benefit of performing a competitive assay.

The stability of liposomes was studied when exposed to the materials and flow rates commonly used in a flow injection apparatus. Liposomes were found to be unstable when exposed to the underivatized glass bead column due to the loss of lipid from the liposome bilayer to hydrophobic sites on the column. Conditioning of the system with lipid prior to the introduction of liposomes helped to stabilize the structures by saturating any exposed hydrophobic sites. The sites remained saturated with continuous use of the system for 2 weeks due to the slow partitioning of the lipid into the aqueous phase. The liposomes were also tested under higher flow rates to determine the degree of rupturing due to shear forces. No leakage of carboxyfluorescein from the structures was observed when exposed to flow rates of 2 mL/min which is typical for use in FIA. The results obtained from injections of liposome samples after conditioning were very reproducible with consistency in dispersion and peak symmetry.

The hydrodynamic characteristics of liposomes were studied extensively to determine the feasibility of using liposomes as analytical reagents in flowing systems. Liposomes are bulky structures in a typical flow injection system with a diameter that corresponds to roughly 1/5000 of the tube diameter. The heterogeneity and the size of the liposomes in solution made it important to determine if the behavior of these structures was consistent with that predicted by the theory developed for a homogeneous solution containing molecules of a similar diffusion coefficient. In theory, the dispersion squared is linearly related to the length of straight tubing through which the sample flows. In a homogeneous solution, the slope of this plot is a constant which is inversely proportional to the square root of the diffusion coefficient. When heterogeneous mixtures are injected into a flowing stream, we can expect some partitioning of the particulates out of the flow stream resulting in uncharacterized dispersions. Plots of carboxyfluorescein-loaded liposomes and carboxyfluorescein solution behavior gave straight lines with correlation coefficients of 0.92 and 0.99, respectively. The ratio of the respective slopes is approximately equal to the inverse ratio of the square root of the diffusion coefficients suggesting that liposome behavior closely follows the behavior of a homogeneous solution. The behavior of liposomes in other common components of a flow injection system, such as a knitted delay tube and a packed reactor column, showed that liposome flow mimicked the theoretical behavior of a homogeneous solution. Therefore, liposome suspensions may be used in FIA as calibration solutions, and so are appropriate analytical reagents in methods of continuous analysis.

## Figures and Tables

**Figure 1:**
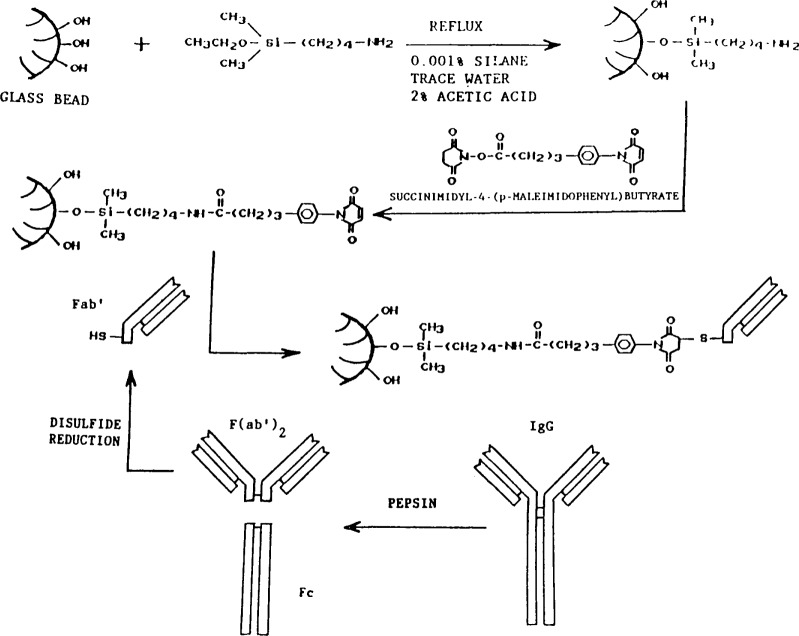
Silanization of nonporous glass bead surface, and covalent attachment of Fab′ fragment.

**Figure 2:**
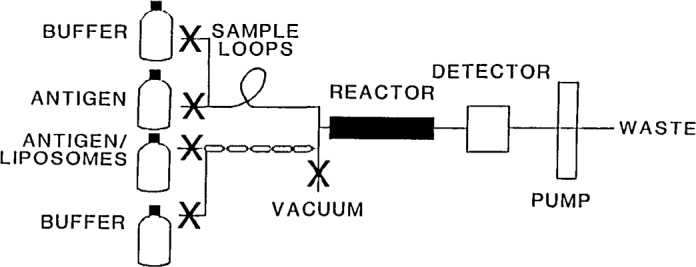
Flow injection system with simultaneous double injection. Xs represent microprocessor-controlled solenoid pinch valves.
